# The relationship between the pan-immune-inflammation value and long-term prognoses in patients with hypertension: National Health and Nutrition Examination Study, 1999–2018

**DOI:** 10.3389/fcvm.2023.1099427

**Published:** 2023-03-02

**Authors:** Bo Wu, Chenlu Zhang, Shuqiong Lin, Yanbin Zhang, Shan Ding, Wei Song

**Affiliations:** ^1^Longyan First Affiliated Hospital of Fujian Medical University, Longyan, China; ^2^Zhangzhou Affiliated Hospital of Fujian Medical University, Zhangzhou, China; ^3^The People’s Hospital of Longyan, Longyan, China

**Keywords:** hypertension, all-cause mortality, cardiovascular mortality, pan-immune-inflammation value, NHANES

## Abstract

**Background:**

Direct antihypertensive therapy in hypertensive patients with a high CVD risk can reduce the incidence of cardiovascular death but increase adverse cardiovascular events, so additional ways to identify hypertensive patients at high risk may be needed. Studies have shown that immunity and inflammation affect the prognoses of patients with hypertension and that the pan-immune-inflammation value (PIV) is an index to assess immunity and inflammation, but few studies have applied the PIV index to patients with hypertension.

**Objective:**

To explore the relationship between the PIV and long-term all-cause and cardiovascular mortality in patients with hypertension.

**Method:**

Data from the National Health and Nutrition Examination Survey (NHANES) 1999–2018 with a mortality follow-up through December 31, 2019, were analyzed. A total of 26,781 participants were evaluated. The patients were grouped based on PIV levels as follows: T1 group (*n* = 8,938), T2 group (*n* = 8,893), and T3 group (*n* = 8,950). The relationship between the PIV and long-term all-cause and cardiovascular death was assessed by survival curves and Cox regression analysis based on the NHANES recommended weights.

**Result:**

The PIV was significantly associated with long-term all-cause and cardiovascular mortality in patients with hypertension. After full adjustment, patients with higher PIV have a higher risk of all-cause [Group 3: HR: 1.37, 95% CI: 1.20–1.55, *p* < 0.001] and cardiovascular [Group 3: HR: 1.62, 95% CI: 1.22–2.15, *p* < 0.001] mortality.

**Conclusion:**

Elevated PIV was associated with increased all-cause mortality and cardiovascular mortality in hypertensive patients.

## Introduction

Hypertension is recognized as one of the major factors affecting the current global burden of disease (1), and the prevalence of hypertension among adults worldwide has been reported to be over 30% and continues to rise (2–4). Epidemiological data show that about 760 million people die from cardiovascular diseases related to high blood pressure each year (5). The 2017 ACC hypertension guidelines recommend antihypertensive therapy for individuals at higher risk for CVD, which will significantly reduce the number of cardiovascular disease events and deaths in the United States but may increase the number of adverse events (6). Therefore, adding other pathways to identify high-risk hypertensive patients may be a viable approach to balancing the distinction between the two.

Recent studies have pointed out that immunity and inflammation are related to the occurrence and development of hypertension. An abnormal immune status will contribute to inflammation and end-organ damage in hypertension (7, 8). In addition, the activation of adaptive immunity can cause inflammation in hypertensive patients, and inflammation can, in turn, promote the occurrence and development of hypertension through the increase in the number of immune cells (9). Moreover, many studies have investigated the relationship between inflammation and the prognoses in patients with hypertension (10, 11). Due to the complex interactions between hypertension and patients’ immune inflammatory responses, metrics based on simple calculations inevitably limit the predictive power of prognosis.

The pan-immune-inflammation value (PIV) is an index that can evaluate the immune and inflammatory status of patients. It incorporates the counts of neutrophils, platelets, monocytes, and lymphocytes. In previous studies, the PIV has been shown to be associated with the prognosis of a variety of diseases related to immunity and inflammation, such as breast cancer (12), antineutrophil cytoplasmic antibody-associated vasculitis (13), and ST-segment elevation myocardial infarction (14). However, few studies have explored the relationship between the PIV and prognoses in hypertensive patients.

The purpose of this study was to explore the relationship between the PIV and long-term all-cause mortality and cardiovascular mortality in community hypertension patients so as to provide insight and suggestions for the treatment and management of hypertension in the American Community.

## Methods

### Study population

The NHANES (National Health and Nutrition Examination Survey) is a research program designed to assess the health and nutrition statuses of adults and children in the United States. The program was started in the early 1960s. It is a survey of different populations and health topics. In 1999, the survey became an ongoing program involving a variety of health and nutrition measurements. Each year, the project surveys a nationally representative sample of approximately 5,000 people. The NCHS ethics review board has approved the NHANES protocol. Written informed consent was obtained from each participant.

The NHANES interview section includes demographic, socioeconomic, diet, and health-related questions. The physical examination includes physiological measurements, laboratory examination results, and so on. A retrospective analysis was performed using publicly available data from the NHANES from 1999 to 2018.

**Figure 1:**
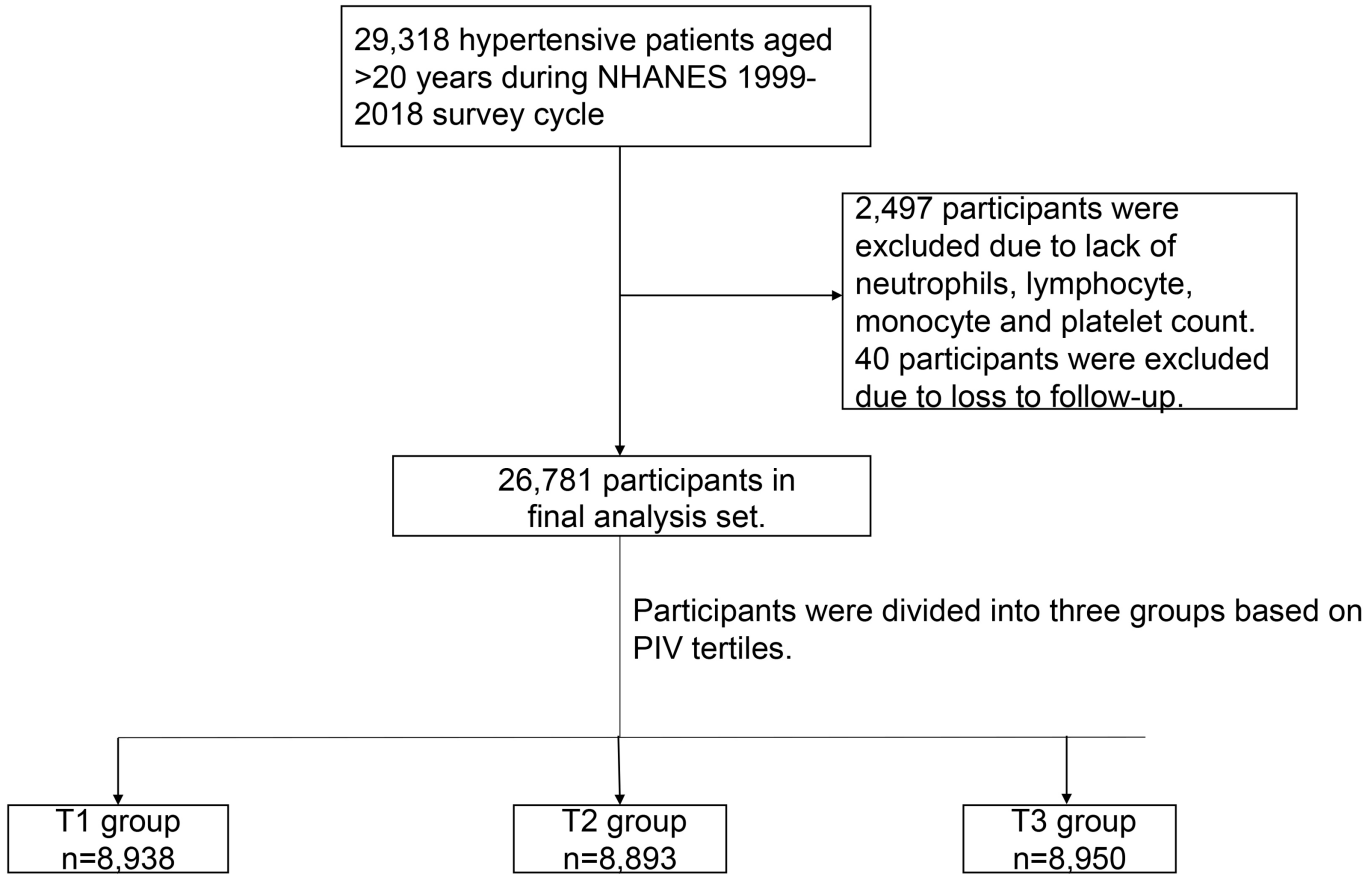
Flowchart of the study design.

In the NHANES 1999–2018, there were a total of 29,318 hypertensive patients aged >20 years and, of them, 2,497 participants were excluded due to a lack of neutrophils, lymphocyte, monocyte, and platelet counts. In addition, 40 participants were excluded due to lack of the data follow-up. Finally, 26,781 participants were enrolled in our study (Figure 1).

### Definition of hypertension

According to the question in the NHANES: “Has a doctor ever told you that you have hypertension?” participants who answered “yes” were considered hypertensive. In addition, according to blood pressure data collected by the NHANES, participants with systolic blood pressure (SBP) >130 mmHg or diastolic blood pressure (DBP) >80 mmHg were considered hypertensive (15). Mean values were used if participants had multiple blood pressure data points. Participants who were taking blood pressure medications were also identified as hypertensive patients.

### Calculation of the PIV

The PIV was calculated as: neutrophil count (10^9^/L) * platelet count (10^9^/L) * monocyte count / Lymphocyte count (10^9^/L). Patients were divided into three groups based on the tertials of the PIV: T1 group (≤199), T2 group (>199 and ≤ 350), and T3 group (>350).

### Primary outcome

The mortality status was determined based on a probabilistic record match with the National Death Index (NDI) using demographic identifiers.^1^ The primary outcome was CVD mortality. The secondary outcome was all-cause mortality. Cause of death was categorized using the International Classification of Diseases, 10th edition (ICD-10). Cardiovascular mortality was categorized using ICD-10 codes I00-I09, I11, I13, and I20-I51. For participants in the NHANES 1999–2018, mortality follow-up data was available through December 31, 2019.

### Definitions of variables of interest

Age, sex, race, and BMI were self-reported by participants. Participants who had smoked more than 100 cigarettes in life or were currently smoking were considered smokers. Participants were considered drinkers if they had consumed an average of one or more drinks per day over the previous 12 months. Metabolic equivalent (MET) were calculated from patients’ self-reported weekly activity time in a manner consistent with previous studies (16). The Healthy Eating Index (HEI-2015) was calculated based on self-reported daily food intake components (17). The diagnosis of comorbidities was based on an affirmative response to the question, “Has a doctor or other health professional ever told you that you had diabetes mellitus (DM), chronic heart failure (CHF), coronary heart disease (CHF), or stroke?” In addition, the participants whose glycohemoglobin HbA1c (%) > 6.5, fasting glucose (mmol/L) ≥ 7.0, random blood glucose (mmol/L) ≥ 11.1, two-hour OGTT blood glucose (mmol/L) ≥ 11.1, or who take diabetes medication or insulin, were also considered to have diabetes. Laboratory measurements, such as creatinine, white blood cells (WBC), hemoglobin (HBG), platelet (PLT), creatinine (Cr), triglycerides (TC) and total cholesterol (TG) were collected using automated hematological analysis equipment. The methods used to derive complete blood count (CBC) parameters are based on the Beckman Coulter method of counting and sizing, in combination with an automatic diluting and mixing device for sample processing, and a single-beam photometer for hemoglobinometry. The white blood count (WBC) differential uses VCS technology. See Chapter 7 of the NHANES Laboratory/Medical Technologists Procedures Manual for details. The NHANES quality control and quality assurance protocols meet the 1988 Clinical Laboratory Improvement Act mandates. Detailed quality control and quality assurance instructions are discussed in the NHANES Laboratory/Medical Technologists Procedures Manual (LPM). The equipment and test methods used in the laboratory tests are provided on the NHANES website.^2^

### Statistical analyses

The analyses were conducted according to NHANES-recommended weights to calculate the weights for specific groups. Continuous variables were expressed as means (standard error), while categorical variables were presented as counts (percentages). Baseline characteristics of the four groups were compared using an analysis of variance (ANOVA) for continuous variables and an *χ*^2^ test for categorical variables.

We used Kaplan–Meier and Cox regression analyses to evaluate the associations between the PIV and long-term all-cause and CVD mortality. Both estimates and probabilities were based on weights recommended by the NHANES. Model 1 was a crude model unadjusted for potential confounders. Model 2 was adjusted for age and gender. Model 3 was fully adjusted for potential confounders, include age, gender, race/ethnicity, smoking status, drinking status, BMI, Cr, TG, TC, HEI-2015, MET, DM, CHD, stroke and CHF. Regression cubic splines was used to explore the potential non-linear relationship between PIV and hypertensive patients. In addition, we explored the relationship between the PIV and all-cause and CVD mortality in different subgroups, including age, sex, obesity, smoking status, drinking status and antihypertensive drug. In addition, Neutrophil to lymphocyte ratio (NLR), as a common inflammatory parameter, was included in the study for additional analysis. Finally, to verify the robustness of the results, the diagnostic criteria for hypertension were set at 140/90 mmHg and sensitivity analysis was performed.

All data analyses were performed by using the Survey package of the R software (Version 4.2.0; R Foundation for Statistical Computing, Vienna, Austria). A two-sided *p*-value <0.05 indicated significance for all analyses.

## Results

### Participant characteristics

In our study, there were a total of 26,781 participants. The average age of the participants was 54.5 (0.2) years old, and they were slightly more likely to be male (51.7%). Participants were divided into three groups based on the tertials of the PIV: T1 group (*n* = 8,938), T2 group (*n* = 8,893), and T3 group (*n* = 8,950). The participants in the group with the highest PIV were older and more likely to be non-Hispanic White 5,127 (77.7%). Compared to the patients in the T1 group, patients in the T3 group had higher BMI [31.01(0.12) kg/㎡], WBC [8.75 (0.03) × 10^9^/L], HB [14.37 (0.03), g/dL], Cr [84.58 (0.64), μmol/L], and TG [1.98 (0.03), mmol/L] values. The proportion of smokers (53.4%) in the T3 group was higher and more likely combined with DM (22.9%), CHF (5.2%), CHD (6.7%), and stroke (6.0%). There were no significant differences among the three groups regarding gender, drinking status, and TC. More data on the baseline characteristics of the study population are detailed in Table 1.

**Table 1:** Baseline study population characteristics (weighted).

variable	Total (*n* = 26,781)	Group 1 (*n* = 8,938)	Group 2 (*n* = 8,893)	Group 3 (*n* = 8,950)	*P*-value
Age, mean (SE), years	54.5(0.2)	53.8(0.3)	54.3(0.2)	55.2(0.3)	<0.001
Male, n (%)	13,843(51.7)	4,508(50.3)	4,596(51.6)	4,739(52.3)	0.141
Race, n (%)					<0.001
Mexican American	4,072(15.2)	1,294(6.6)	1,451(6.3)	1,327(5.9)	
Non-Hispanic Black	6,291(23.5)	3,178(20.6)	1,791(10.3)	1,322(7.3)	
Non-Hispanic White	12,298(45.9)	2,893(59.8)	4,278(72.5)	5,127(77.7)		
Other Hispanic	1,970(7.4)	685(4.9)	661(4.5)	624(4.4)	
Other Race	2,150(8)	888(8.0)	712(6.4)	550(4.8)	
WBC, mean (SE), 1,000/μL	7.39(0.03)	6.09(0.04)	7.11(0.02)	8.75(0.03)	<0.001
HB, mean (SE), g/dL	14.35(0.02)	14.25(0.03)	14.42(0.03)	14.37(0.03)	<0.001
PLT, mean (SE), 1,000/μL	253.22(0.78)	217.09(0.84)	249.56(0.88)	286.78(1.21)	<0.001
Neutrophil, mean (SE), 1,000/μL	4.41(0.02)	3.11(0.02)	4.16(0.02)	5.73(0.02)	<0.001
Lymphocyte, mean (SE), 1,000/μL	2.14(0.01)	2.30(0.03)	2.14(0.01)	2.02(0.01)	<0.001
Monocytes, mean (SE), 1,000/μL	0.58(0.00)	0.46(0.00)	0.56(0.00)	0.71(0.00)	<0.001
BMI, mean (SE), kg/㎡	30.35(0.07)	29.63(0.10)	30.31(0.12)	31.01(0.12)	<0.001
Cr, mean (SE), μmol/L	82.24(0.33)	81.01(0.43)	80.88(0.42)	84.58(0.64)	<0.001
TG, mean (SE), mmol/L	1.91(0.02)	1.80(0.02)	1.95(0.02)	1.98(0.03)	<0.001
TC, mean (SE), mmol/L	5.17(0.01)	5.19(0.02)	5.18(0.02)	5.15(0.02)	0.086
HEI-2015, mean (SE),	50.62(0.17)	51.39(0.25)	51.09(0.24)	49.52(0.24)	<0.001
MET, mean (SE), minutes/week	3,146.75(65.92)	3,404.04(110.36)	3,065.76(81.31)	2,998.98(112.88)	0.010
Hypotensive drugs, n (%)	14,791(86.15)	4,783(82.05)	4,822(82.49)	5,186(85.86)	<0.001
Smoke, n (%)	13,179(49.2)	4,028(45.5)	4,327(48.7)	4,824(53.4)	<0.001
Alcohol*, n (%)	15,294(57.1)	5,064(63.9)	5,175(65.6)	5,055(63.0)	0.056
DM, n (%)	6,944(25.9)	2,237(18.9)	2,288(20.1)	2,419(21.9)	<0.001
CHF, n (%)	1,443(5.4)	376(3.2)	446(3.9)	621(5.2)	<0.001
CHD, n (%)	1,785(6.7)	468(4.7)	616(5.9)	701(6.7)	<0.001
Stroke, n (%)	1,684(6.3)	481(4.2)	516(4.4)	687(6.0)	<0.001
All-cause death, n (%)	6,229(18.0)	1,600(14.4)	1,959(16.1)	2,670(22.8)	<0.001
CVD death, n (%)	1,721(5.1)	429(3.9)	536(4.4)	756(6.7)	<0.001

*The average number of drinks consumed per day over the past 12 months. WBC, White blood cells; HB, hemoglobin; PLT, platelets; BMI, body mass index; Cr, creatinine; TG, triglyceride; TC, total cholesterol; HEI-2015, Healthy Eating Index-2015; Met, metabolic equivalent; DM, diabetes mellitus; CHF, chronic heart failure; CHD, CVD, Cardiovascular disease.

### All-cause mortality and CVD mortality

**Figure 2:**
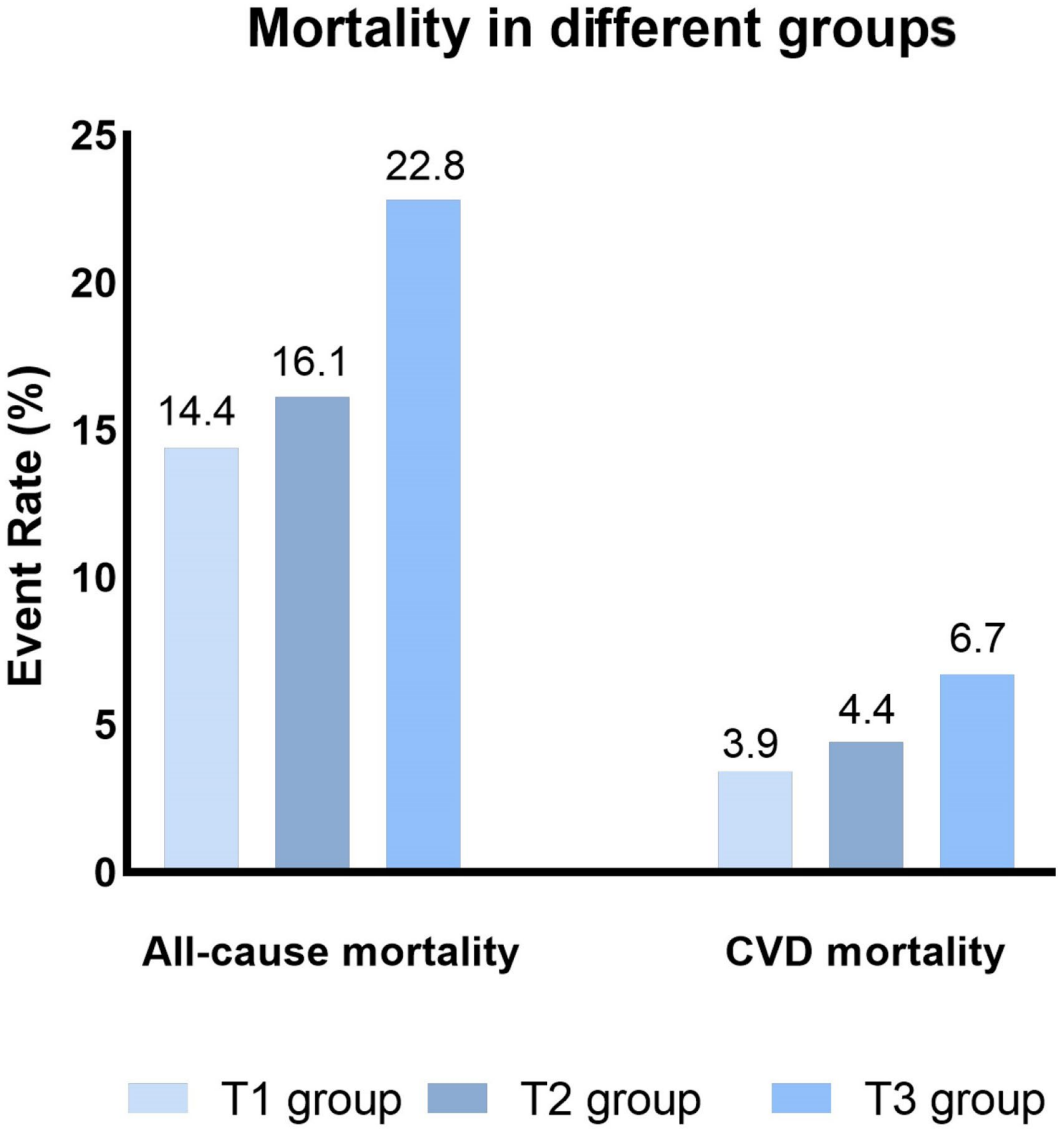
Kaplan–Meier survival estimates for long-term all-cause and CVD mortality (weighted).

**Figure 3:**
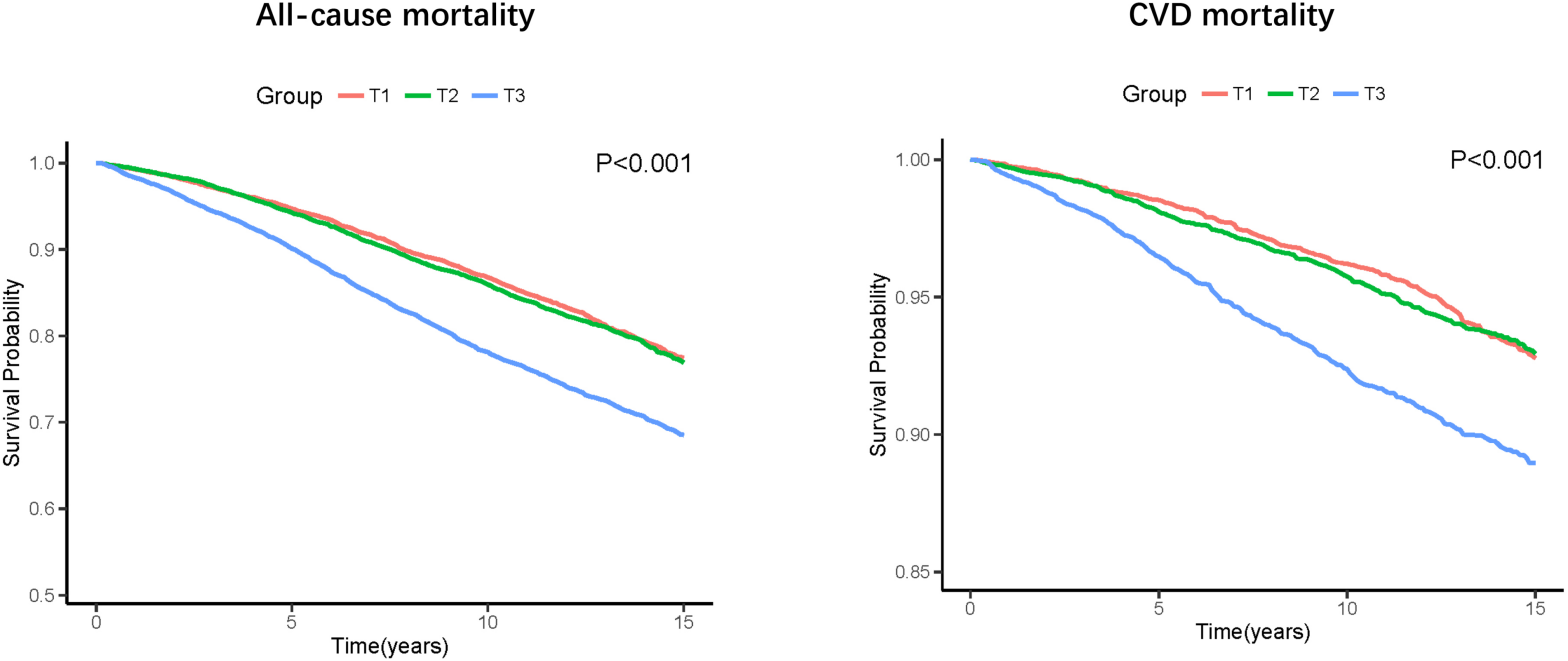
The all-cause and CVD mortality in different groups (weighted).

During a median follow-up of 8.75 years, 6,229 (18.0%) all-cause death and 1,721 (5.1%) CVD deaths occurred. As shown in Figure 2, the T3 group had the highest all-cause and CVD mortality. Kaplan–Meier survival analysis curves also showed that the T3 group had the highest all-cause and CVD mortality (P-log rank <0.001, Figure 3). The univariate Cox proportional risk analysis showed that the T3 group has a higher risk of all-cause (HR: 1.59, 95% CI: 1.46–1.73; *p* < 0.001) and CVD (HR: 1.75, 95% CI: 1.48–2.08; *p* < 0.001) death (Model 1). After full adjusted for age, gender, race, smoking status, drinking status, TG, TC, HEI-2015, MET, BMI, Cr, CHF, CHD, DM, and stroke, compared to the T1 group, the T3 groups has a higher risk of all-cause (HR: 1.37, 95% CI: 1.25–1.50; *p* < 0.001) and CVD (HR: 1.56, 95% CI: 1.30–1.87; *p* < 0.001) death (Table 2). In addition, the results were similar to those of PIV after regression analysis by grouping the NLR tertiles (eTable 1).

**Table 2:** The association between PIV and all-cause and CVD mortality.

	Model 1		Model 2		Model 3	
	HR (95%CI)	*P*-value	HR (95%CI)	*P*-value	HR (95%CI)	*P*-value
All-cause death						
Group 1	ref		ref		ref	
Group 2	1.05 (0.97–1.15)	0.244	0.98 (0.91–1.06)	0.622	0.95 (0.84–1.07)	0.387
Group 3	1.59 (1.46–1.73)	<0.001	1.40 (1.30–1.51)	<0.001	1.37 (1.20–1.55)	<0.001
CVD death						
Group 1	ref		ref		ref	
Group 2	1.06 (0.87–1.30)	0.571	0.99 (0.84–1.18)	0.950	1.04 (0.81–1.32)	0.766
Group 3	1.75 (1.48–2.08)	<0.001	1.56 (1.34–1.82)	<0.001	1.62 (1.22–2.15)	<0.001

Model 1: Not adjusted. Model 2: Adjusted by age, gender. Model 3: Adjusted by age, gender, race/ethnicity, smoking status, drinking status, BMI, Cr, TG, TC, HEI-2015, MET, DM, CHD, stroke, CHF.

### Sensitivity analysis

After defining patients with blood pressure above 140/90mmHg as hypertensive, 21,270 people were enrolled, with 5,676 all-cause deaths and 1,603 CVD deaths. The results of COX regression analysis were consistent with the previous results. In unadjusted model, compared to the T1 group, the T3 groups has a higher risk of all-cause (HR: 1.59, 95% CI: 1.44–1.75; *p* < 0.001) and CVD (HR: 1.72, 95% CI: 1.44–2.05; *p* < 0.001) death. In full adjusted model, the T3 groups has a higher risk of all-cause (HR: 1.35, 95% CI: 1.19–1.54; *p* < 0.001) and CVD (HR: 1.55, 95% CI: 1.19–2.03; *p* < 0.001) death (eTable 2).

### Subgroup analysis

#### All-cause death in different subgroups

**Figure 4:**
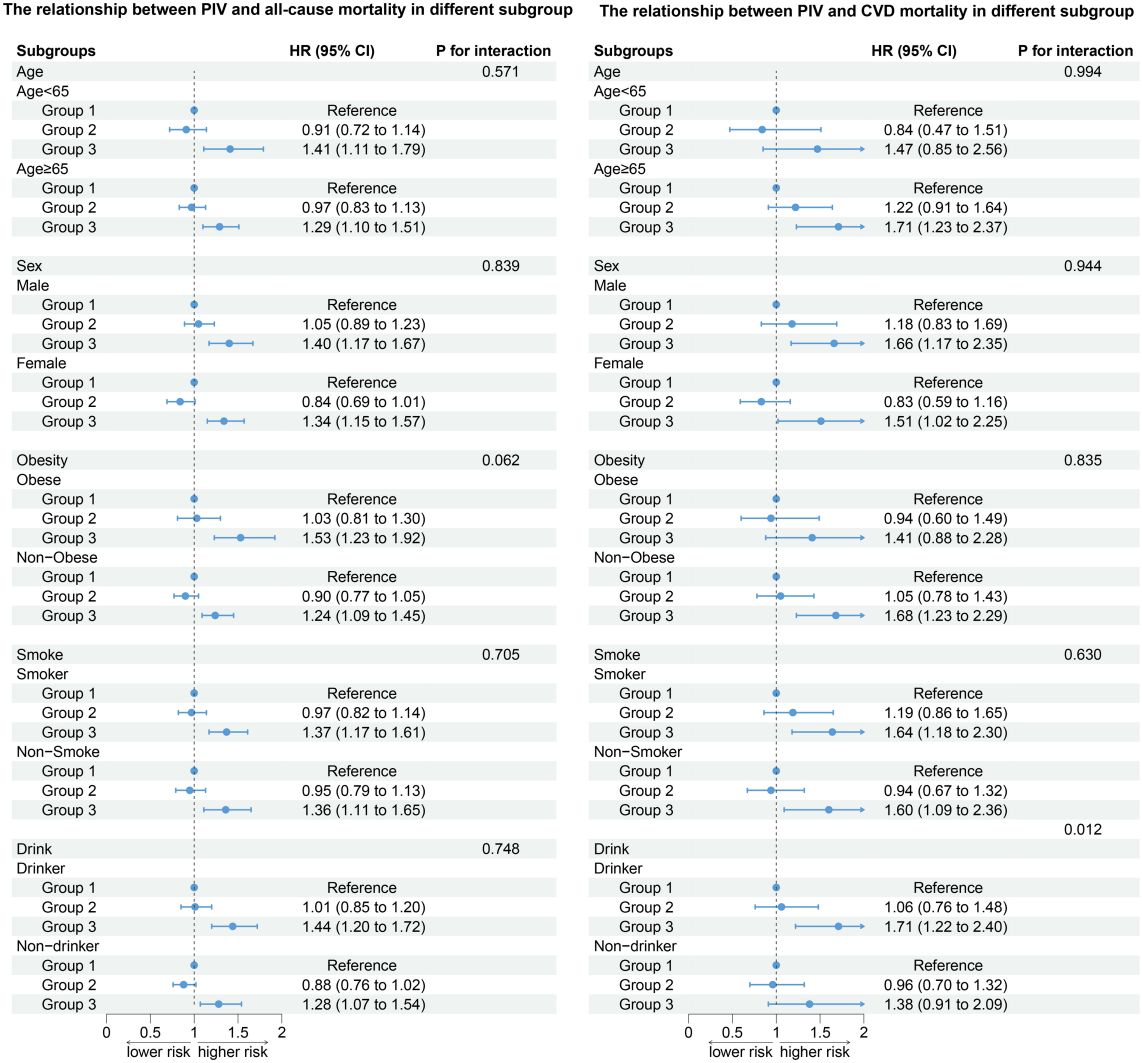
Association between PIV and all-cause and CVD mortality by selected subgroups (weighted).

**Figure 5:**
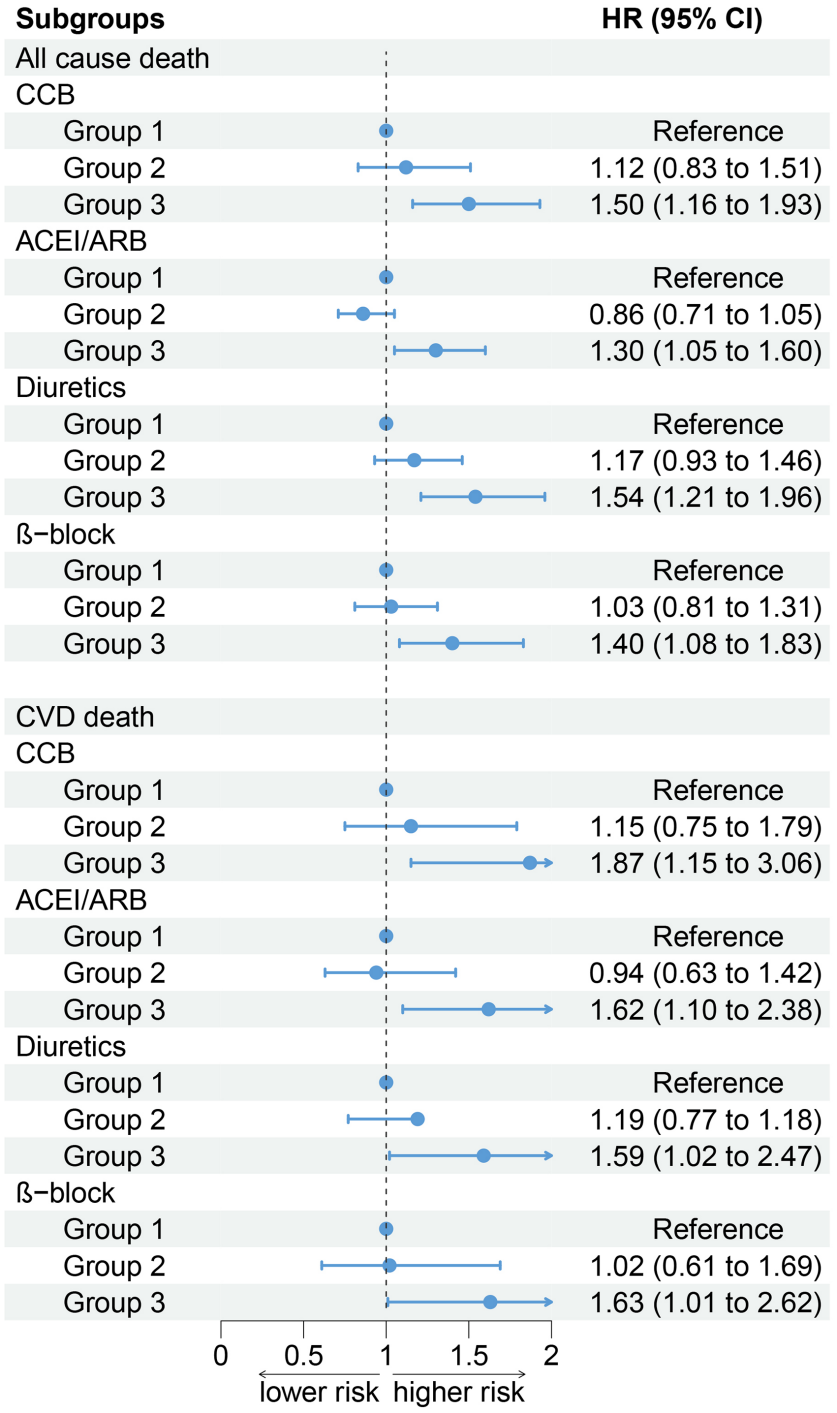
Association between PIV and all-cause and CVD mortality by hypotensive drugs stratification (weighted).

After stratifying the participants according to age, gender, obesity, smoking status, and drinking status, the association between PIV and all-cause death (age: P for interaction = 0.571; gender: P for interaction = 0.839; obesity: P for interaction = 0.062; smoke: P for interaction = 0.705; drink: P for interaction = 0.748) did not change. Compared to the T1 group, the T3 group has a higher risk of all-cause death (Figure 4). The results of stratified analysis according to drug use are shown in Figure 5.

#### CVD death in different subgroups

After stratifying the participants according to age, gender, obesity, and smoking status, the association between the PIV and CVD death (age: P for interaction = 0.994; gender: P for interaction = 0.944; obesity: P for interaction = 0.835; smoke: P for interaction = 0.630) did not change. Participants in the T3 group have a higher risk of all-cause and CVD death compared to the T1 group. In addition, our results show that there was an interaction between the PIV and drinking (non-drinkers: HR: 1.73, 95% CI: 1.22–2.44; Drinker: HR: 1.80, 95% CI: 1.27–2.55, P for interaction = 0.012). A high PIV was not significantly associated with the risk of CVD death in non-drinkers (Figure 4). The results of stratified analysis according to drug use are shown in Figure 5

### Regression cubic splines

**Figure 6:**
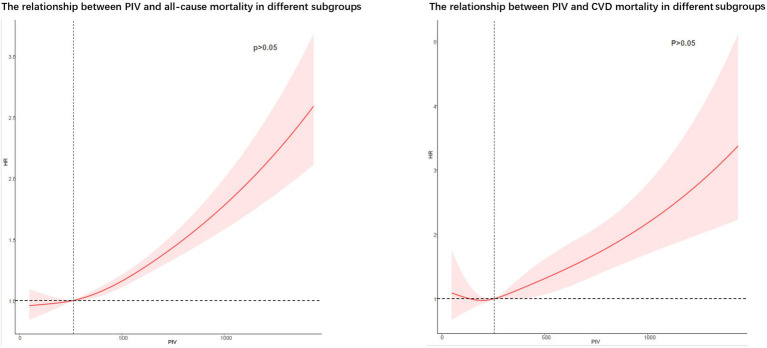
Potential non-linear relationships between PIV and all-cause and cardiovascular death (weighted).

RCS showed no potential non-linear relationship between PIV and all-cause and CVD death (Figure 6).

## Discussion

In this retrospective study, our results showed that the PIV was associated with prognoses in hypertensive patients. A high PIV level is an independent risk factor for all-cause death and CVD mortality in hypertensive patients.

One-third of adults worldwide are reported to have hypertension, and the prevalence continues to rise (18). As a major risk factor for cardiovascular diseases, hypertension claims 10 million lives worldwide each year (19). Studies have shown that 45% of deaths from ischemic heart disease and 51% of deaths from cerebrovascular disease can be attributed to hypertension (20). In the face of such a high morbidity and mortality, screening and early intervention for hypertensive patients at risk for poor prognoses are critical. The 2017 ACC Hypertension Guidelines recommend antihypertensive therapy for individuals at higher risk for CVD, which could significantly reduce the number of cardiovascular disease events and deaths in the United States but may increase the number of adverse events (6). Therefore, adding other pathways to identify high-risk hypertensive patients may be a viable way to balance the distinction between the two.

Research suggests that hypertension is an immune and inflammatory disease (21). Blood pressure was found association with immune cells, and that lymphocytes may increase blood pressure by regulating vascular function, sympathetic efflux, and renal sodium reabsorption and salt treatment by antigen presenting cells (22–24). In addition, some studies have found that people with hypertension have elevated levels of circulating inflammatory cytokines, including IL-6, IL-1β, and TNF-α (22). Elevated pro-inflammatory factors will promote the progression of inflammation, increase endothelial damage and vascular remodeling damage, increase arterial stiffness, and lead to increased blood pressure (25). Interesting, antigen-presenting cells can activate T-effector lymphocytes and lead to low-grade inflammation, which can lead to increased blood pressure and organ damage (8, 21, 26). In addition, chronic inflammation can promote a thrombotic state, thus increasing the risk of cardiovascular death in hypertensive patients (27). Although many studies have also shown that inflammation is a risk factor for poor prognoses in hypertensive patients (10, 28), few studies have combined inflammatory and immune responses to assess outcomes in hypertensive patients. Patients’ immune response, as a key factor in the inflammatory state of hypertension (29), should also be considered when evaluating the effects of inflammation on the prognosis of hypertensive patients.

The PIV is an index that combines neutrophils, platelets, monocytes, and lymphocytes and has been considered a comprehensive assessment of the immune and inflammatory statuses in patients in previous studies (30). Previous studies have further indicated that pancreatic cancer patients with high PIV levels before radiotherapy had worse prognoses (31). Lee et al. noted that patients with neutrophil cytoplasmic antibody-associated vasculitis with a high PVI had lower long-term survival (13). In a prognostic study of patients with ST-segment elevation MI, the PIV also proved to be a useful predictor of long-term survival (14). The association of hypertension with immune response and inflammation led us to associate the PIV with the prognosis of hypertension. In our study, all-cause mortality and cardiovascular mortality were significantly higher in the group with the highest PIV levels than in the other three groups, and the cox regression analysis showed that a high PIV level was an independent risk factor for long-term mortality in hypertensive patients, which is consistent with previous studies (13, 14, 30, 31). In addition, considering that setting the diagnostic criteria for hypertension at 130/80 mmHg might overestimate the number of patients with hypertension, we performed a sensitivity analysis by setting the diagnostic criteria at 140/90 mmHg. The results of sensitivity analysis were consistent with the main results. After adjusting for possible influencing factors, only patients in group 3 had an increased risk of all-cause CVD death. This makes our results more reliable.

The association between the PIV and all-cause mortality in hypertensive patients did not change after stratification for age, sex, obesity, smoking status, and alcohol consumption. The risk of long-term all-cause mortality was significantly higher in hypertensive patients with high PIV levels than in those in the other three groups. However, our results showed that high levels of PIV did not affect the risk of CVD death in hypertensive patients who did not drink alcohol. Although the specific mechanism of the interaction between drinking and PIV remains unclear, some previous studies may help explain our results. Several studies have shown that alcohol consumption amplifies the effects of inflammation. For example, studies by Yu Sun et al. point out that alcohol can sensitizes Kupffer cells to TNF-α Over-production (25). Alcohol exposure can cause a decrease in peripheral B cells and the ability to produce protective antibodies (32). In addition, alcohol exposure increases the expression of proinflammatory cytokines and, thus, leads to inflammation-mediated tissue damage (33). The effects of alcohol on the gut cannot be ignored either. Excessive drinking can encourage bacteria, or bacterial products, to leak from the gut into the bloodstream, triggering a range of inflammatory and immune responses (34).These negative effects of alcohol are associated with an increased risk of cardiovascular death (35–37).

In addition, we further analyzed the effect of NLR on cardiovascular death. In contrast to PIV, platelet and monocyte were not included in the calculation of NLR. Our results found that NLR without platelets and monocytes appeared to be more sensitive to cardiovascular death. But we think that PIV is more appropriate for prognostic assessment of cardiovascular death in the community population. First of all, surface molecules on platelets can interact with other cells and chemokines in activated platelets and cause inflammation thrombosis events and CVD (38). In addition, monocyte infiltration is also one of the main culprits of arterial plaque formation (39, 40). In a study of older adults in a Korean community, monocytes were found to be the best predictor of CVD death (41).

Our findings further suggest that High levels of PIV are associated with poor outcomes in hypertensive patients. PIV is an indicator that can be calculated from simple blood parameters and can be used as a biomarker of poor prognosis in hypertensive patients. In addition, adding the PVI to further comprehensive assessments of hypertensive patients at high risk of CVD and treating these patients with antihypertensive therapy may be an effective way to effectively balance cardiovascular events with other adverse events.

### Limitations

As a retrospective study, our study has some limitations. First, the diagnosis of hypertension and comorbidities is partly based on the self-reports of patients, which may deviate from the actual disease situation. Second, all baseline data were obtained by one blood draw and questionnaire survey, and the patients’ data may change during the long-term follow-up. Third, due to the limitations of the retrospective analysis, we were unable to prove a causal relationship between the PIV and death in hypertensive patients, and further prospective studies are, therefore, needed to validate our conclusions. Fourth, our database is limited by the public database, and some data is missing. For example, detailed data on immune diseases, anti-inflammatory drugs and C-reactive protein.

## Conclusion

The PIV is an independent risk factor for all-cause mortality and cardiovascular mortality in hypertensive patients. This may provide a theoretical basis for future research on effective ways to include further comprehensive assessments of the PIV in high-risk hypertensive patients with CVD to balance cardiovascular events with other adverse events.

## Data availability statement

The raw data supporting the conclusions of this article will be made available by the authors, without undue reservation.

## Ethics statement

Ethical review and approval was not required for the study on human participants in accordance with the local legislation and institutional requirements. Written informed consent for participation was not required for this study in accordance with the national legislation and the institutional requirements.

## Author contributions

WS designed the research, is the guarantor of this work, had full access to all of the data used in the study, and takes responsibility for the integrity of the data and the accuracy of the data analysis. BW performed the analyses and wrote the first draft of the manuscript. CZ, SL, YZ, SD, and WS revised the manuscript. All authors read and approved the final manuscript and its submission.

## Funding

This research was funded and supported by Longyan City Science and Technology Plan Project (2022LYF17026).

## Conflict of interest

The authors declare that the research was conducted in the absence of any commercial or financial relationships that could be construed as a potential conflict of interest.

## Publisher’s note

All claims expressed in this article are solely those of the authors and do not necessarily represent those of their affiliated organizations, or those of the publisher, the editors and the reviewers. Any product that may be evaluated in this article, or claim that may be made by its manufacturer, is not guaranteed or endorsed by the publisher.

## Supplementary material

The Supplementary material for this article can be found online at: https://www.frontiersin.org/articles/10.3389/fcvm.2023.1099427/full#supplementary-material

## Footnotes

^1^ http://www.cdc.gov/nchs/data/datalinkage/nh99+_mortality_matching_methodology_final.pdf

^2^ https://www.cdc.gov/nchs/nhanes/index.htm
