# Supplementary material for: The relationship between the pan-immune-inflammation value and long-term prognoses in patients with hypertension: National Health and Nutrition Examination Study, 1999–2018
Source: Front Cardiovasc Med. 2023 Mar 2;10:1099427. doi: 10.3389/fcvm.2023.1099427 (PMC10017977; doi:10.3389/fcvm.2023.1099427)
Supplement: Supplementary file 1 [file Table_1.docx]

**eTable 1. The association between NLR and all-cause and CVD mortality.**

|  | Event/All population | | | | | model 1 | | | | model 2 | | | | model 3 | | |
| --- | --- | --- | --- | --- | --- | --- | --- | --- | --- | --- | --- | --- | --- | --- | --- | --- |
|  | |  | HR (95%CI) | | | | P-value | | HR (95%CI) | | P-value | | HR (95%CI) | | | P-value |
| All-cause death | |  | | |  | | |  | |  | |  | | |  | |
| Group 1 | | 1,600/9,234 | ref | | | |  | | ref | |  | | ref | | |  |
| Group 2 | | 1,959/9,029 | | 1.20(1.10-1.31) | | | <0.001 | | 1.08(1.00-1.17) | | 0.061 | | 0.98(0.86-1.16) | | | 0.773 |
| Group 3 | | 2,670/8,518 | | 2.12(1.93-2.32) | | | <0.001 | | 1.59(1.48-1.71) | | <0.001 | | 1.41(1.25-1.58) | | | <0.001 |
| CVD death | |  | |  | | |  | |  | |  | |  | | |  |
| Group 1 | | 383/9,234 | | ref | | |  | | ref | |  | | ref | | |  |
| Group 2 | | 521/9,029 | | 1.42(1.22-1.67) | | | <0.001 | | 1.25(1.08-1.44) | | <0.001 | | 1.32(1.03-1.70) | | | 0.027 |
| Group 3 | | 817/8,518 | | 2.80(2.39-3.29) | | | <0.001 | | 2.02(1.76-2.33) | | <0.001 | | 1.80(1.41-2.30) | | | <0.001 |

Model 1: Not adjusted.

Model 2: Adjusted by age, gender.

Model 3: Adjusted by age, gender, race/ethnicity, smoking status, drinking status, BMI, Cr, TG, TC, HEI-2015, MET, DM, CHD, stroke, CHF.
