# Supplementary material for: The relationship between the pan-immune-inflammation value and long-term prognoses in patients with hypertension: National Health and Nutrition Examination Study, 1999–2018
Source: Front Cardiovasc Med. 2023 Mar 2;10:1099427. doi: 10.3389/fcvm.2023.1099427 (PMC10017977; doi:10.3389/fcvm.2023.1099427)
Supplement: Supplementary file 2 [file Table_2.docx]

**eTable 2. The association between PIV and all-cause and CVD mortality in patients with hypertension(140/90mmHg)**

|  | Event/All population | | | | | model 1 | | | | model 2 | | | | model 3 | | |
| --- | --- | --- | --- | --- | --- | --- | --- | --- | --- | --- | --- | --- | --- | --- | --- | --- |
|  | |  | HR (95%CI) | | | | P-value | | HR (95%CI) | | P-value | | HR (95%CI) | | | P-value |
| All-cause death | |  | | |  | | |  | |  | |  | | |  | |
| Group 1 | | 1,472/ 7,090 | ref | | | |  | | ref | |  | | ref | | |  |
| Group 2 | | 1,808/ 7,086 | | 1.01(0.98-1.18) | | | 0.134 | | 1.02(0.94-1.11) | | 0.585 | | 0.98(0.83-1.07) | | | 0.350 |
| Group 3 | | 2,396/ 7,094 | | 1.59(1.44-1.75) | | | <0.001 | | 1.46(1.37-1.59) | | <0.001 | | 1.35(1.19-1.54) | | | <0.001 |
| CVD death | |  | |  | | |  | |  | |  | |  | | |  |
| Group 1 | | 406/7,090 | | ref | | |  | | ref | |  | | ref | | |  |
| Group 2 | | 507/7,086 | | 1.05(0.87-1.28) | | | 0.600 | | 1.03(0.87-1.22) | | 0.759 | | 0.97(0.76-1.21) | | | 0.772 |
| Group 3 | | 690/7,094 | | 1.72(1.44-2.05) | | | <0.001 | | 1.65(1.40-1.95) | | <0.001 | | 1.55(1.19-2.03) | | | <0.001 |

Model 1: Not adjusted.

Model 2: Adjusted by age, gender.

Model 3: Adjusted by age, gender, race/ethnicity, smoking status, drinking status, BMI, Cr, TG, TC, HEI-2015, MET, DM, CHD, stroke, CHF.
